# miR-589-5p inhibits MAP3K8 and suppresses CD90^+^ cancer stem cells in hepatocellular carcinoma

**DOI:** 10.1186/s13046-016-0452-6

**Published:** 2016-11-11

**Authors:** Xi Zhang, Peng Jiang, Ling Shuai, Kai Chen, Zhonghu Li, Yujun Zhang, Yan Jiang, Xiaowu Li

**Affiliations:** Institute of Hepatobiliary Surgery, Southwest Hospital, Third Military Medical University, Chongqing, 400037 China

**Keywords:** Cellular heterogeneity, Tumorigenicity, CD90, microRNA, MAP3K8, Prognosis

## Abstract

**Background:**

Cancer stem cells (CSCs) are important in the tumorigenesis and progression of hepatocellular carcinoma (HCC). MicroRNAs (miRNAs) play crucial roles regulating CD133^+^ and EpCAM^+^ CSCs in HCC, although it is unclear whether miRNAs regulate CD90^+^ CSCs in HCC.

**Methods:**

The miRNA profiles of CD90^+^ and CD90^-^ HCC cells were analyzed using a miRNA microarray and quantitative real-time PCR (qRT-PCR). CSC characteristics were examined by qRT-PCR and Western blot of pluripotency-associated genes, clone and sphere formation assay, transwell migration assay, and nude mice tumorigenicity assay. miR-589-5p mimic transfection was used to overexpress miR-589-5p *in vitro*. The CD90 and miR-589-5p expressions of HCC samples were detected by immunohistochemistry and qRT-PCR, respectively.

**Results:**

miR-589-5p and miR-33b-5p were down-regulated in CD90^+^ cells. Overexpression of miR-589-5p suppressed CD90^+^ CSC characteristics such as Oct4, Sox2 and Nanog expression, a high likelihood of forming cell spheres, high invasiveness and high tumorigenicity. Luciferase reporter assays demonstrated that miR-589-5p directly binds to the 3ˈ-untranslated region of mitogen-activated protein kinase kinase kinase 8 (MAP3K8) mRNA, and exogenous miR-589-5p down-regulated MAP3K8 expression. In addition, siRNA inhibition of MAP3K8 also suppressed CD90^+^ CSC characteristics, even in the absence of miR-589-5p overexpression. In HCC tissues, miR-589-5p expression was inversely correlated with CD90 expression, and high CD90 expression and low miR-589-5p expression were positively correlated with vascular invasion and recurrence and significantly decreased disease-free and overall survival by clinical analysis.

**Conclusion:**

In HCC, miR-589-5p down-regulates the stemness characteristics of CD90^+^ CSCs in part by silencing MAP3K8. CD90 and miR-589-5p expression predict HCC outcomes and might be novel molecular targets for HCC treatment.

**Electronic supplementary material:**

The online version of this article (doi:10.1186/s13046-016-0452-6) contains supplementary material, which is available to authorized users.

## Background

Hepatocellular carcinoma (HCC), which includes over 90 % of liver cancers, is the leading cause of cancer mortality worldwide [1]. Because HCC is resistant to chemoradiotherapy and has a high recurrence and metastatic rate after surgery, HCC patients have a very low five-year survival rate [2, 3]. In recent years, accumulating evidence has suggested that in HCC, a population of cells with stem cell-like features, known as cancer stem cells (CSCs) or tumor-initiating cells (TICs), is essential for the recurrence, metastasis and resistance to chemoradiotherapy seen in HCC [4]. Previous studies have shown that CD90, EpCAM, CD133, CD24, OV-6 and CD44 can be used as CSC markers in HCC, and cells expressing these markers possessed CSC characteristics such as self-renewal, tumor generation and aggressive growth [5–10]. However, the optimal marker for identifying CSC populations in HCC remains disputed.

MicroRNAs (miRNAs) are a large group of small, noncoding RNAs that are important regulators of post-transcriptional gene expression. miRNAs bind to the 3'-untranslated region (UTR) of the target mRNA by base pairing and suppress protein expression through translational repression or mRNA degradation [11–13]. miRNAs play key roles in a variety of physiological and pathological processes, including embryo development, cellular differentiation, stem cell self-renewal and tumor progression [14]. In human solid tumors, many miRNAs have been found to participate in CSC maintenance [15–17]. To date, in HCC, miR-130b has been shown to promote CD133^+^ CSC tumorigenicity and self-renewal [18], whereas miR-181 inhibition reduces the number of EpCAM^+^ CSCs and tumor-initiating ability [19]. However, it remains unclear which miRNA regulates the stemness of CD90^+^ HCC CSCs.

Mitogen-activated protein kinase kinase kinase 8 (MAP3K8), also known as tumor progression locus 2 (TPL2) or cancer Osaka thyroid (COT), is a member of the serine/threonine protein kinase family. MAP3K8 activation is critically involved in inflammation and has variable effects on tumors [20, 21]. In lung and intestinal cancer, MAP3K8 is a tumor suppressor gene [22–24]. However, MAP3K8 is predominantly considered a tumor-promoting oncogene in several tumor types, such as breast cancer, colon cancer, renal cell carcinoma, endometrial cancer, gastric cancer, nasopharyngeal carcinoma, thymoma and lymphoma [20]. MAP3K8 is up-regulated in multiple tumor types and is closely related to tumorigenesis and/or cancer progression [25–27]. However, the role of MAP3K8 in HCC initiation and progression remains unknown. In this study, we found that miR-589-5p was down-regulated in CD90^+^ CSCs and examined the effects of miR-589-5p expression and its target protein MAP3K8 on CD90^+^ CSCs and the clinical outcomes of HCC.

## Methods

### Patients and tissue specimens

Tumor specimens were obtained from 2006 to 2008. All of the patients underwent surgical resection of primary, pathologically confirmed HCC at the Institute of Hepatobiliary Surgery, Southwest Hospital, Third Military Medical University. The tumor stage was based on the Edmondson grade [28]. Sixty-six formalin-fixed, paraffin-embedded tumor specimens were used for IHC, and forty frozen tumor specimens were used for RNA extraction. All of the patients were followed for 5 years.

### Cell lines and culture

For routine culture, MHCC97H, MHCC97L and HepG2 HCC cell lines purchased from the Shanghai Cell Collection (Shanghai, China) were maintained in high-glucose DMEM (Gibco, 8113035) containing 10 % FBS in a 5 % CO_2_ incubator at 37 °C, whereas the SMMC-7721 cell line was maintained in RPMI medium 1640 (Gibco, 8112340). Both media contained 10 % fetal bovine serum (FBS, Gibco, 10099141), penicillin (500 U/ml) and streptomycin (500 μg/ml).

### Flow cytometry

Cells isolated from HCC cell lines, spheres and tumor xenografts were labeled with anti-human antibodies at 4 °C for 20 minutes according to the manufacturer’s instructions (antibodies are described in Additional file 1: Table S1). For unconjugated primary antibodies, the cells were subsequently incubated with FITC- or PE-conjugated secondary antibodies at 4 °C for 20 minutes. The labeled cells were detected using a FACSAria II system (BD Biosciences). The FcR Blocking Reagent (Miltenyi, 130-059-901) was used to increase antibody specificity.

### Cell sorting

The MHCC97H and MHCC97L HCC cells were dissociated into single cells and labeled with anti-CD90 antibodies at 4 °C for 20 minutes. Subsequently, the cells were magnetically labeled with anti-mouse IgG1 MicroBeads (Miltenyi) according to the manufacturer’s instructions. In brief, the cell suspension was loaded onto a MACS column, which was placed in the magnetic field of a MACS Separator. The unlabeled cells (CD90^-^ cells) passed through the column, and the magnetically labeled cells (CD90^+^ cells) were retained. After removing the column from the magnetic field, the magnetically retained CD90^+^ cells were eluted.

### miRNA microarray

The miRNA expression profiles of CD90^+^ and CD90^-^ cells isolated from the HCC cell lines MHCC97H and MHCC97L were compared using a 5th generation miRCURY LNA™ microRNA Array (v.14.0) (Exiqon). RNA extraction, RNA quality control, RNA labeling, array hybridization and data analysis were performed at the Kangchen Biotechnology Corporation (Shanghai, China). Then, the scanned images were imported using GenePix Pro 6.0 software (Axon).

### Quantitative real–time PCR

For qRT-PCR of mRNA targets, total RNA was extracted from cancer cells using RNAiso Plus (TaKaRa, 09108B). cDNA synthesis was performed according to the manufacturer’s instructions (TaKaRa, DRR047A), and qRT-PCR was performed with SYBR Premix Ex Taq II (TaKaRa, DRR081A) using a LightCycler system (Roche). The PCR reaction conditions for all of the assays were 94 °C for 30 seconds, followed by 40 cycles of amplification (94 °C for 5 seconds, 60 °C for 30 seconds and 72 °C for 30 seconds). GAPDH mRNA was used to normalize RNA inputs. The qRT-PCR primers are listed in Additional file 1: Table S2.

For qRT-PCR of miRNAs, small RNAs were extracted from cancer cells or tumor tissues using RNAiso for small RNAs (TaKaRa, D340A). miRNAs were converted to cDNA using a cDNA synthesis kit (TaKaRa, DRR047A), and qRT-PCR was performed with SYBR Premix Ex Taq II (TaKaRa, DRR081A) using a LightCycler system (Roche). The PCR reaction conditions for all of the assays were 95 °C for 20 seconds, followed by 40 cycles of amplification (95 °C for 10 seconds, 60 °C for 20 seconds and 70 °C for 5 seconds). U6 was used to normalize the RNA inputs. All of the primers were from the Bulge-Loop™ miRNA qRT-PCR primer set (RiboBio, MQP-0102, China).

### Luciferase gene reporter

The *in silico* predictions of the potential binding regions in MAP3K8 mRNA for miRNAs were performed using TargetScan (http://www.targetscan.org). Wild-type and mutant MAP3K8 3′-UTR luciferase plasmids were generated using the pmiR-RB-REPORT™ vector (RiboBio, China). The full-length wild-type MAP3K8 3′-UTR is 1463 bp. The wild type sense sequence was 5′-GATATGCACC GGTCTCAAGG TTCTCATTTC-3′, and the mutant sense sequence was 5′- GATATGCACC GGTCTCAAGG AAGACATTTC-3′. Exponentially growing 293 T cells were transfected with wild-type or mutant vectors using Lipofectamine® 2000 reagent (Invitrogen, 11668027, USA) according to the manufacturer's instructions. The miR-589-5p mimics or non-target control (RiboBio, NC#22, China) were co-transfected with the vectors for 48 hours, and then luciferase activity was measured.

### Clone and sphere formation assay

For the clone formation assay, 500 cells were sorted by MACS and seeded per well in 6-well plates. After 10 days of culture, the clones were fixed using methanol and dyed with hematoxylin, and the number of clones (>50 cells) was assessed microscopically.

For the sphere formation assay, 1000 cells were sorted by MACS and seeded per well in ultra-low attachment 6-well plates (Costar, 3741). The cells were cultured in DMEM/F12 media (Sigma) containing B27 supplement (Gibco, 17504-044), antibiotics, 20 ng/ml EGF (Peprotech, AF-100-15) and 20 ng/ml bFGF (Peprotech, 100-18B). Fresh medium was added every 3-5 days. After 2 weeks of culture, spheres with a diameter >75 μm were counted. For FACS analysis, the spheres were collected and dissociated into single cells using trypsin.

### Cell invasion and migration assays

The invasion and migration assays were performed in 24-well Millicell hanging inserts (Millipore) with or without a Matrigel layer (BD Biosciences) according to the manufacturer's instructions. Briefly, 1 × 10^5^ cells were seeded into the top chamber, and DMEM with 10 % FBS was added to the bottom chamber as a chemoattractant. After a 48 hour incubation at 37 °C, the numbers of cells that invaded the Matrigel (invasion) or membrane (migration) were counted in 10 fields using a 40× objective lens.

### Tumor formation in nude mice

To assess tumor formation in nude mice, CD90^+^ and CD90^-^ cells were sorted and injected (amounts ranging from 1 × 10^3^ to 5 × 10^5^) subcutaneously into different sides of 6-week-old male nude mice for controlled visualization and comparison. The mice were maintained under standard conditions and were examined for tumor formation for 12 weeks. After the tumors formed, the mice were sacrificed, and xenografts were harvested for IHC and primary culture. The fresh tumor xenografts from the nude mice were cut into small pieces and plated in a cell culture flask, and tumor cells migrated out from these pieces. DMEM containing 15 % FBS was used to initially establish the primary cultures, and DMEM containing 10 % FBS was used for subsequent maintenance.

To assess the effect of miR-589-5p on HCC tumorigenesis, 3 days after 1 × 10^5^ CD90^+^ MHCC97H cells were subcutaneously injected into nude mice, micrON™ agomir-589-5p (25 nmol, 50 μl) or control RNAs (RiboBio, China) were injected into the same site every 3 days within the next 2 weeks. The mice were maintained under standard conditions and were examined for tumor formation for 12 weeks.

### miR-589-5p mimic/antagomir transfection

The miR-Ribo™ miR-589-5p mimic/antagomir and negative control miRs are commercially available (RiboBio, China), and the experiments were performed according to the manufacturer's instructions. In brief, 5 × 10^5^ cells were seeded per well in 6-well plates. The miR-589-5p mimics/antagomir (or control miRs) and Lipofectamine® 2000 were diluted in Opti-MEM® (Gibco, 31985-062, USA) separately, were mixed gently and were added to the culture plates. The final concentration of mimic was 50 nM, and the final concentration of antagomir was 100 nM. After a 24 hour incubation at 37 °C, the cells were used for additional experiments.

### siRNA transfection

The siRNAs and negative control RNAs were synthesized and purified by Sangon Biotech (Shanghai, China). Synthesized siRNAs were transfected into sorted CD90^+^ MHCC97H and MHCC97L cells with Lipofectamine® 2000 according to the manufacturer’s protocol. The siRNAs for MAP3K8 were sense: 5′-GCGCCTTTGGAAAGGTATATT-3′ and antisense: 5′-TATACCTTTCCAAAGGCGCTT-3′. The negative control siRNAs were sense: 5′-TTCTCCGAACGTGTCACGTTT-3′ and antisense: 5′-ACGTGACACGTTCGGAGATT-3′. The final concentration of siRNAs was 25 nM. After a 24 hour incubation at 37 °C, the cells were used for further experiments.

### Western blot analysis

Prepared cells were lysed in radioimmunoprecipitation assay (RIPA) buffer supplemented with a protease inhibitor (Roche, Branford, CT). Total proteins (30 μg/well) were separated by electrophoresis on 12 % sodium dodecyl sulfate-polyacrylamide gels. Subsequently, protein samples were transferred onto nitrocellulose membranes (Pierce, Thermo Fisher Scientific, Waltham, MA) and incubated with corresponding primary antibodies (antibodies are described in Additional file 1: Table S1). The membranes were incubated with horseradish peroxidase-conjugated secondary antibodies and developed using SuperSignal™ chemiluminescence reagent (Pierce, Thermo Fisher Scientific) according to the manufacturer’s instruction. Protein expression levels were normalized against GAPDH.

### Immunohistochemistry

HCC, paired non-tumor tissues and tumor xenografts from nude mice were fixed with formalin and embedded in paraffin. Then, samples were sectioned (5 μm) and attached to poly-L-lysine coated slides. The slides were deparaffinized, treated with 3 % H_2_O_2_ at 37 °C for 1 hour to block endogenous peroxidase activity, and heated in 10 mM citrate buffer at 120 °C for 2 min and 10 sec for antigen retrieval. After incubation with the primary antibody (antibodies are described in Additional file 1: Table S1) at 4 °C overnight, the samples were incubated with a secondary peroxidase-conjugated antibody for 60 min at 37 °C and then developed with DAB (Dako, 00080066). Hematoxylin was used as a counterstain. The staining intensity of CD90 was described as low or high according to the percentage of positively stained cells. Because CD90 is expressed at a basal level in most liver cells, only cells expressing a significantly higher level of CD90 were considered positive [29].

### Statistics

The data were analyzed using SPSS 18 software (SPSS Corp., Chicago, IL, USA) and are presented as the mean values ± standard error of mean (SEM) or the median with the range. When two groups were compared, Student’s t test or the Mann-Whitney U test was used. Chi-square or Fisher’s exact methods were used for clinical statistical analyses. The Kaplan–Meier and Cox regression methods were used for survival analyses. *p* < 0.05 was considered statistically significant and is indicated by *. *p* < 0.01 was considered highly statistically significant and is indicated by **. Experiments were performed in three independent repeats in triplicate.

## Results

### miR-589-5p is down-regulated in CD90^+^ HCC cells

In HCC, several molecules, including CD90, CD133, CD24, OV-6, EpCAM and CD44, have been used as potential CSC markers for primary tumors or cell lines [5–10]. An ideal CSC marker should be expressed in all primary tumors and cell lines and should be stably expressed in a small cell population. Using flow cytometric analysis, we observed that in all of the tested HCC cell lines (MHCC97H, MHCC97L, HepG2 and SMMC-7721), CD90 was consistently expressed in a small population (0.9 % to 3.1 %, Additional file 2: Figure S1A), even under cell sphere formation conditions, which increased in all four cell lines up to 11.8 % (Additional file 2: Figure S1B). However, the expressions of other markers, such as EpCAM and CD133, were verified in different cell lines (Additional file 2: Figure S1A). We further confirmed that CD90^+^ HCC cells exhibited CSC characteristics, such as pluripotency-associated gene (Oct4, Sox2 and Nanog) expression, a greater likelihood to form cell spheres, a high invasiveness and high tumorigenicity (Additional file 2: Figure S2). Thus, CD90 might be an ideal marker to identify the CSC population in HCC.

**Fig. 1:**
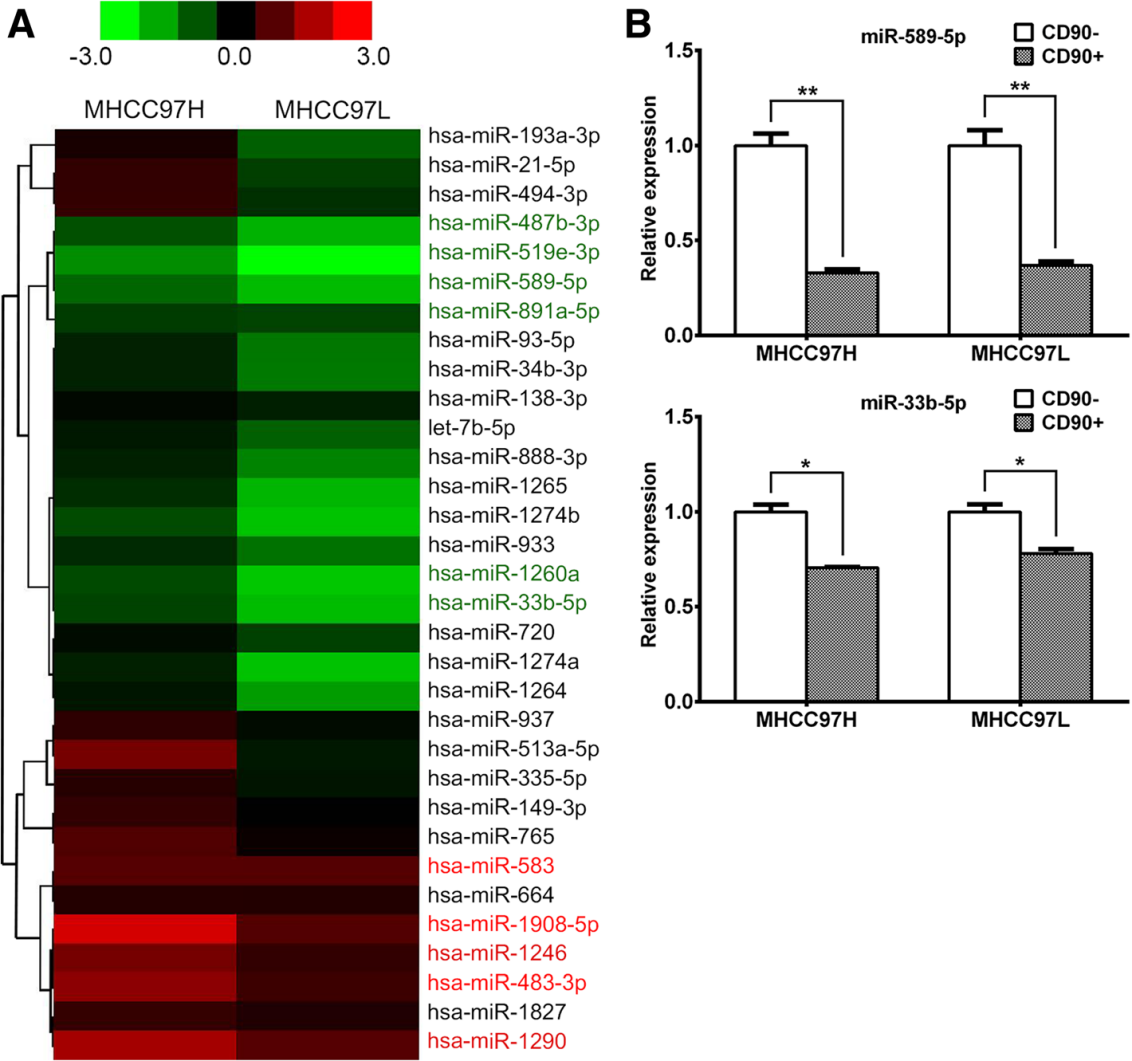
miR-589-5p is down-regulated in CD90^+^ HCC cells. (**a**) The results of miRNA microarrays were shown as hierarchical clustering of miRNAs that are differentially expressed in CD90^+^ HCC cells. The color scale illustrates the relative expression level of a miRNA: red color represents a high expression level; green color represents a low expression level. (**b**) Expression of miR-589-5p and miR-33b-5p in both CD90^+^ HCC cell lines was determined using qRT-PCR. All data are representative of three independent experiments and are shown as mean ± SEM (n = 3)

It has been reported that miRNAs play very important roles maintaining the stemness of CSCs [16]. Therefore, we used a miRNA microarray to examine the miRNA expression profiles in MHCC97H and MHCC97L CD90^+^ and CD90^-^ cells. As shown in Additional file 1: Table S3, eleven up-regulated and ten down-regulated miRNAs were found in MHCC97H CD90^+^ cells compared with MHCC97H CD90^-^ cells, and thirteen up-regulated and twenty-two down-regulated miRNAs were found in the MHCC97L CD90^+^ cells compared with the MHCC97L CD90^-^ cells. Combining the two data sets, a total of eleven miRNAs with altered expression, five of which were up-regulated and six of which were down-regulated, were observed in both of the CD90^+^ HCC cell lines (Fig. 1a).

To confirm the miRNA microarray results, we used quantitative real-time PCR (qRT-PCR) to demonstrate that these miRNAs were expressed differently in CD90^+^ and CD90^-^ cells from the MHCC97H and MHCC97L cell lines. We found that the expression of miR-589-5p and miR-33b-5p were down-regulated, which were the only miRNAs that agreed with the miRNA microarray results (Fig. 1b). Because miR-589-5p showed a more significant fold change than miR-33b-5p, we focused on miR-589-5p in further studies.

### miR-589-5p helps regulate the stemness of HCC CSCs

**Fig. 2:**
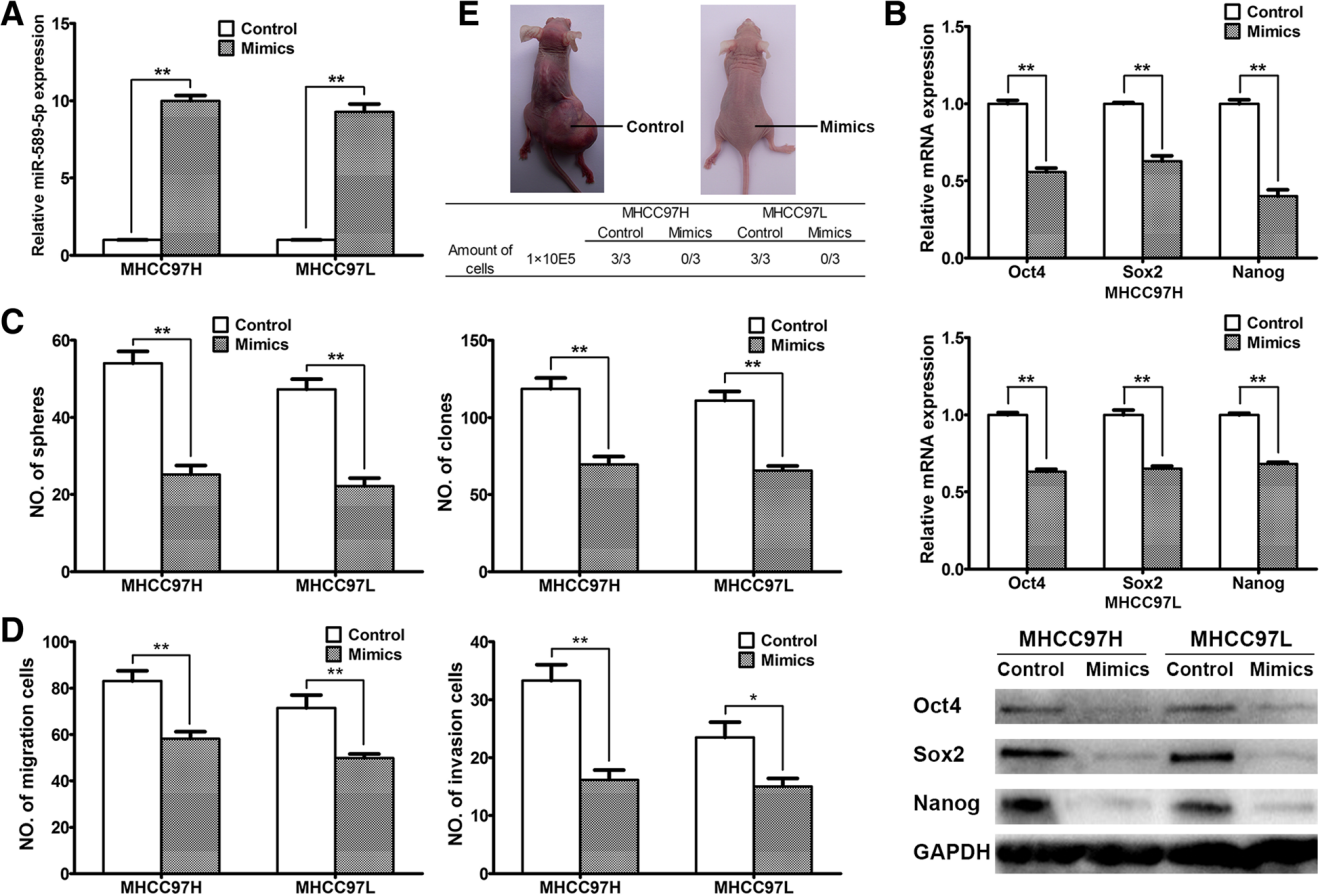
miR-589-5p participates in the regulation of stemness in HCC CSCs. (**a**) Expression of miR-589-5p increased in CD90^+^ MHCC97H and MHCC97L cells after transfection with miR-589-5p mimics. (**b**) Expression of the pluripotency-associated genes Oct4, Sox2 and Nanog in CD90^+^ MHCC97H and MHCC97L cells was measured by qRT-PCR (upper two panels) and Western blot (lower panel) after transfection of miR-control or miR-589-5p mimics. (**c**) Sphere formation and clone formation by CD90^+^ MHCC97H and MHCC97L cells transfected with miR-control or miR-589-5p mimics. (**d**) Migration and invasion of CD90^+^ MHCC97H and MHCC97L cells transfected with miR-control or miR-589-5p mimics. (**e**) Tumor formation by CD90^+^ MHCC97H and MHCC97L cells in nude mice. After the tumor cells were injected, the miR-589-5p agomirs or control RNAs were injected subcutaneously every three days within the next two weeks. All data are representative of three independent experiments and are shown as mean ± SEM (n = 3)

Next, we determined whether miR-589-5p participates in regulating the stemness of HCC CSCs. The sorted CD90^+^ MHCC97H and MHCC97L cells were transfected with miR-589-5p mimics for 24 hours to overexpress miR-589-5p (Fig. 2a), and cells transfected with miR-589-5p mimics exhibited lower levels of Oct4, Sox2 and Nanog compared to the negative control RNA (Fig. 2b). Moreover, the ability of the cells to form cell spheres and the migration of MHCC97H and MHCC97L were greatly reduced compared to the controls (Fig. 2c, 2d). However, overexpression of miR-589-5p had no impact on the regulation of stemness in CD90^-^ HCC cells (Additional file 2: Figure S3A-3D).

To determine the impact of miR-589-5p on the tumorigenic capacity of CD90^+^ cells, sorted CD90^+^ cells were subcutaneously injected into nude mice. As shown in Fig. 2e, after 1 × 10^5^ CD90^+^ cells were injected into the mice, micrON™ agomir-589-5p or control RNAs were subsequently injected to overexpress miR-589-5p or as a control. The mice injected with control RNAs initiated tumors (3/3 mice) within 12 weeks, whereas no tumor formation was observed in mice injected with the miR-589-5p mimic, suggesting that miR-589-5p suppresses the CD90^+^ CSC characteristics both *in vitro* and *in vivo*.

### miR-589-5p directly targets MAP3K8

**Fig. 3:**
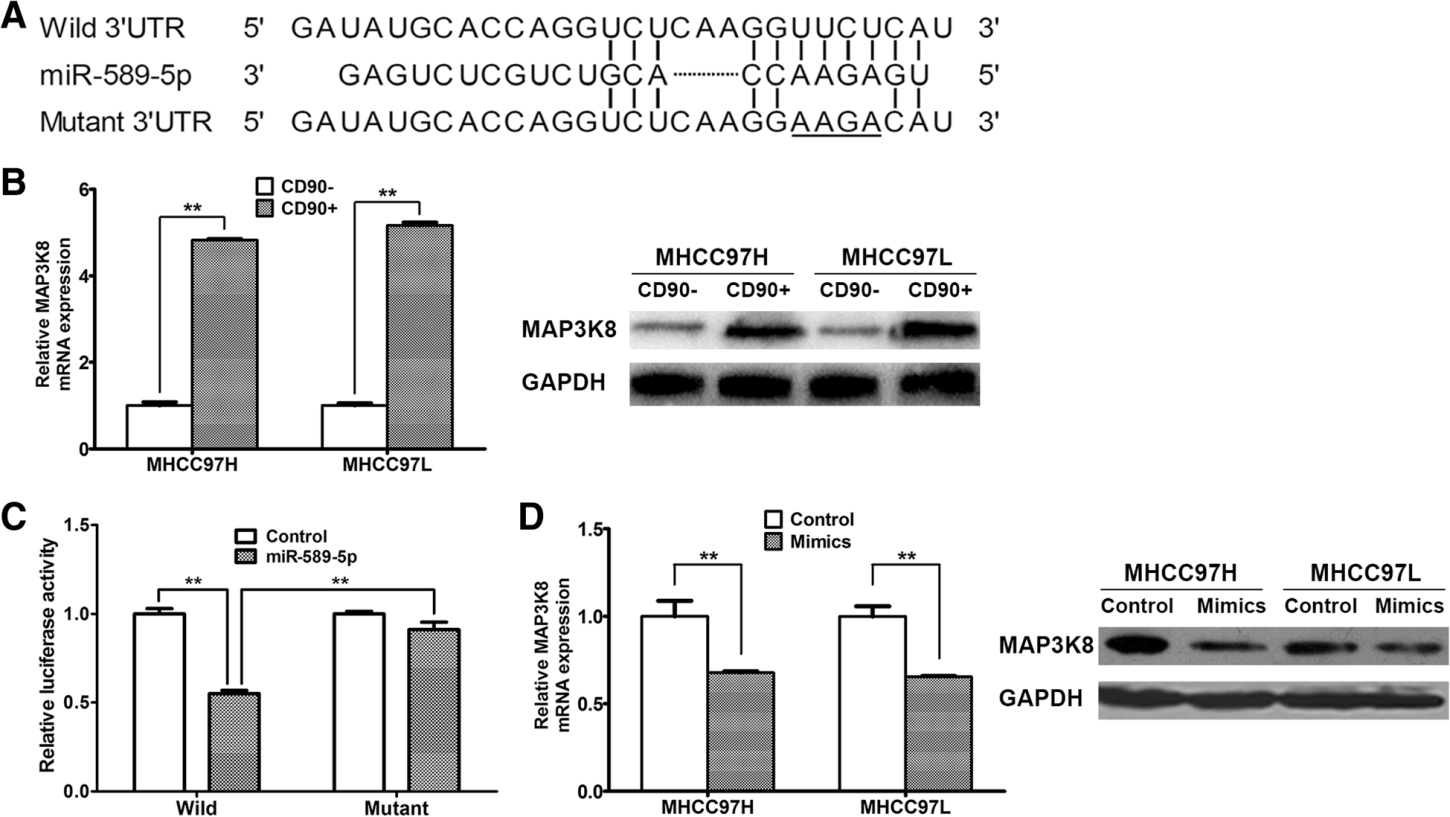
miR-589-5p directly targets MAP3K8. (**a**) The potential binding sites for miR-589-5p in the MAP3K8 mRNA 3ˈ-UTR were determined with *in silico* predictions. The underlined bases were mutated to AAGA in the mutant plasmid. (**b**) The expression of MAP3K8 in CD90^+^ and CD90^-^ cells sorted from MHCC97H and MHCC97L cells measured by qRT-PCR and Western blot. (**c**) Luciferase activities of MAP3K8 wild-type (Wild) and mutant reporter plasmids in 293 T cells co-transfected with miR-control or miR-589-5p. (**d**) CD90^+^ MHCC97H and MHCC97L cells were transfected with miR-589-5p mimics for 24 hours, and the level of MAP3K8 expression was measured by qRT-PCR and Western blot. All data are representative of three independent experiments and are shown as mean ± SEM (n = 3)

To investigate how miR-589-5p affects the stemness of CSCs, we used *in silico* predictions to identify miR-589-5p targets. We identified MAP3K8 as a potential miR-589-5p target; MAP3K8 is a known tumor-promoting gene in various human tumors. The 3ˈ- UTR of the MAP3K8 mRNA has a binding site for miR-589-5p, and this binding site is conserved among different species, including humans, chimps and mice (Fig. 3a). Thus we examined the expression of MAP3K8 in CD90^+^ and CD90^-^ cells sorted from MHCC97H and MHCC97L cells. Figure 3b showed that the level of MAP3K8 expression was higher in CD90^+^ cells than in CD90^-^ cells at both the mRNA and protein levels.

To determine whether MAP3K8 is a direct target of miR-589-5p, full-length human MAP3K8 mRNA 3′-UTR luciferase reporter vectors (wild-type and mutant plasmids, as described in the Materials and Methods, Fig. 3a) were co-transfected into 293 T cells with miR-589-5p or miR-control. Cells co-transfected with miR-589-5p and the wild-type MAP3K8 3′-UTR exhibited a 45.1 % reduction in luciferase activity compared to the miR-control. In contrast, the mutant MAP3K8 3′-UTR showed a 93.8 % restoration of luciferase (Fig. 3c), suggesting that miR-589-5p directly binds the 3'-UTR of the MAP3K8 mRNA.

To investigate the influence of miR-589-5p on MAP3K8 expression, we transfected miR-589-5p mimics into sorted CD90^+^ and CD90^-^ MHCC97H and MHCC97L cells. The expression of MAP3K8 was decreased at both the mRNA and protein levels in CD90^+^ and CD90^-^ cells (Fig. 3d and Additional file 2: Figure S3E), indicating that miR-589-5p directly targets and decreases the expression of MAP3K8 by binding its 3′-UTR.

### miR-589-5p inhibits the stemness of HCC CSCs through MAP3K8

**Fig. 4:**
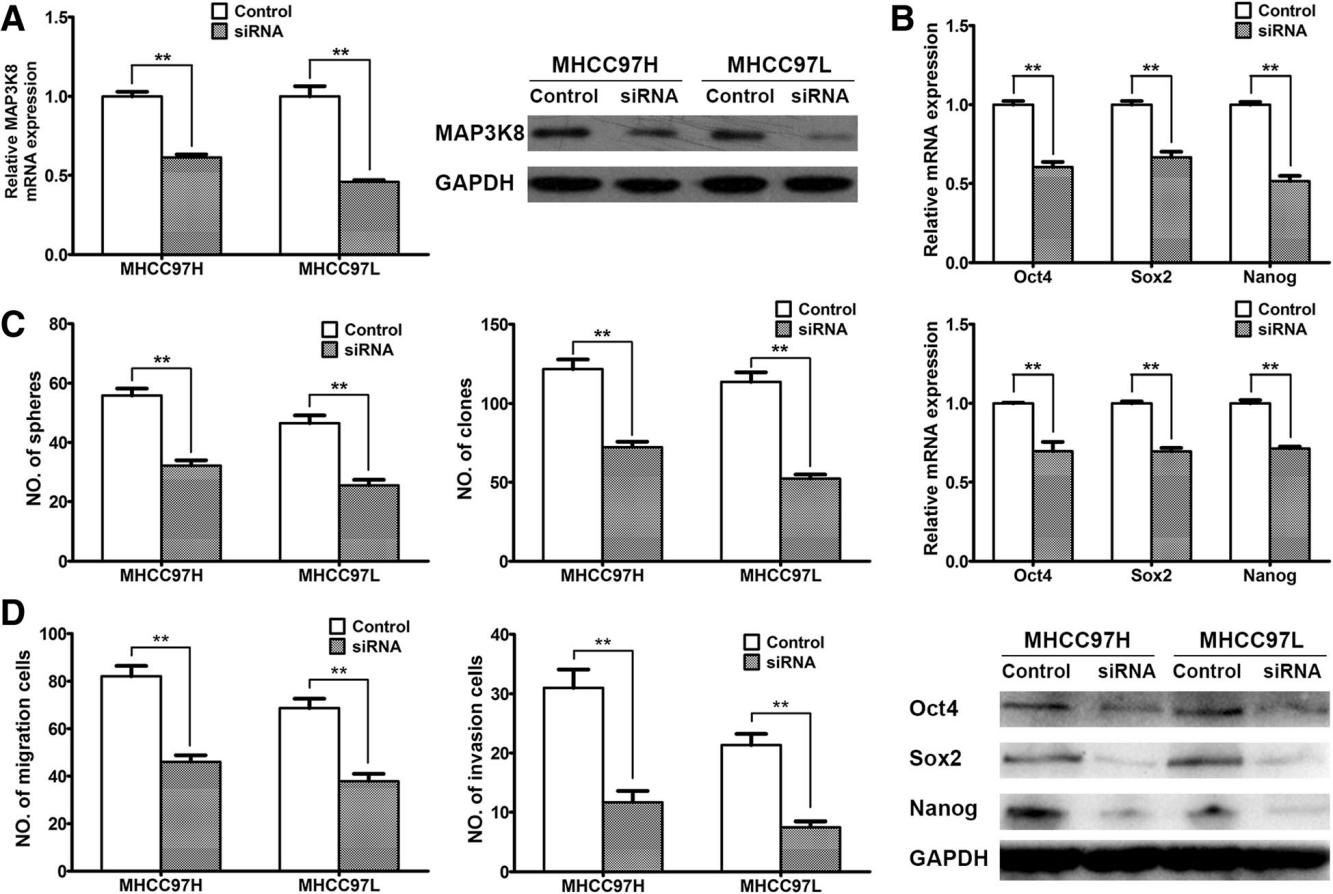
miR-589-5p inhibits the stemness of HCC CSCs through MAP3K8. (**a**) CD90^+^ MHCC97H and MHCC97L cells were transfected with MAP3K8 siRNA, and the mRNA and protein levels of MAP3K8 both decreased. (**b**) Expression of the pluripotency-associated genes Oct4, Sox2 and Nanog in CD90^+^ MHCC97H and MHCC97L cells was measured by qRT-PCR (upper two panels) and Western blot (lower panel) after transfection of MAP3K8 siRNA or negative control RNA. (**c**) Sphere formation and clone formation by CD90^+^ MHCC97H and MHCC97L cells transfected with MAP3K8 siRNA or negative control RNA. (**d**) Migration and invasion of CD90^+^ MHCC97H and MHCC97L cells transfected with MAP3K8 siRNA or negative control RNA. All data are representative of three independent experiments and are shown as mean ± SEM (n = 3)

To examine the significance of MAP3K8 to HCC stemness, an siRNA targeting MAP3K8 was transfected into CD90^+^ cells sorted from MHCC97H and MHCC97L. qRT-PCR and Western blot analyses showed that MAP3K8 expression was markedly repressed after siRNA transfection at both the mRNA and protein levels, respectively (Fig. 4a). The pluripotency-associated genes were also repressed in the siRNA group compared with the control group (Fig. 4b).

To confirm the role of MAP3K8 in the tumorigenic and metastatic potential of HCC cells, sphere and clone formation and cell migration were evaluated in sorted CD90^+^ MHCC97H and MHCC97L cells after MAP3K8 siRNA transfection. Compared to the negative control, down-regulation of MAP3K8 by siRNA significantly reduced the ability of cells to form spheres and clones and also suppressed cell migration and invasion in transwell assays (Fig. 4c, 4d). These data indicate that siRNA inhibition of MAP3K8 also suppressed the stemness of CD90^+^ CSCs even in the absence of miR-589-5p overexpression, and miR-589-5p likely functions through MAP3K8 to suppress CSC stemness and HCC progression.

### The expression of CD90 and miR-589-5p is associated with a poor clinical prognosis in human HCC

**Fig. 5:**
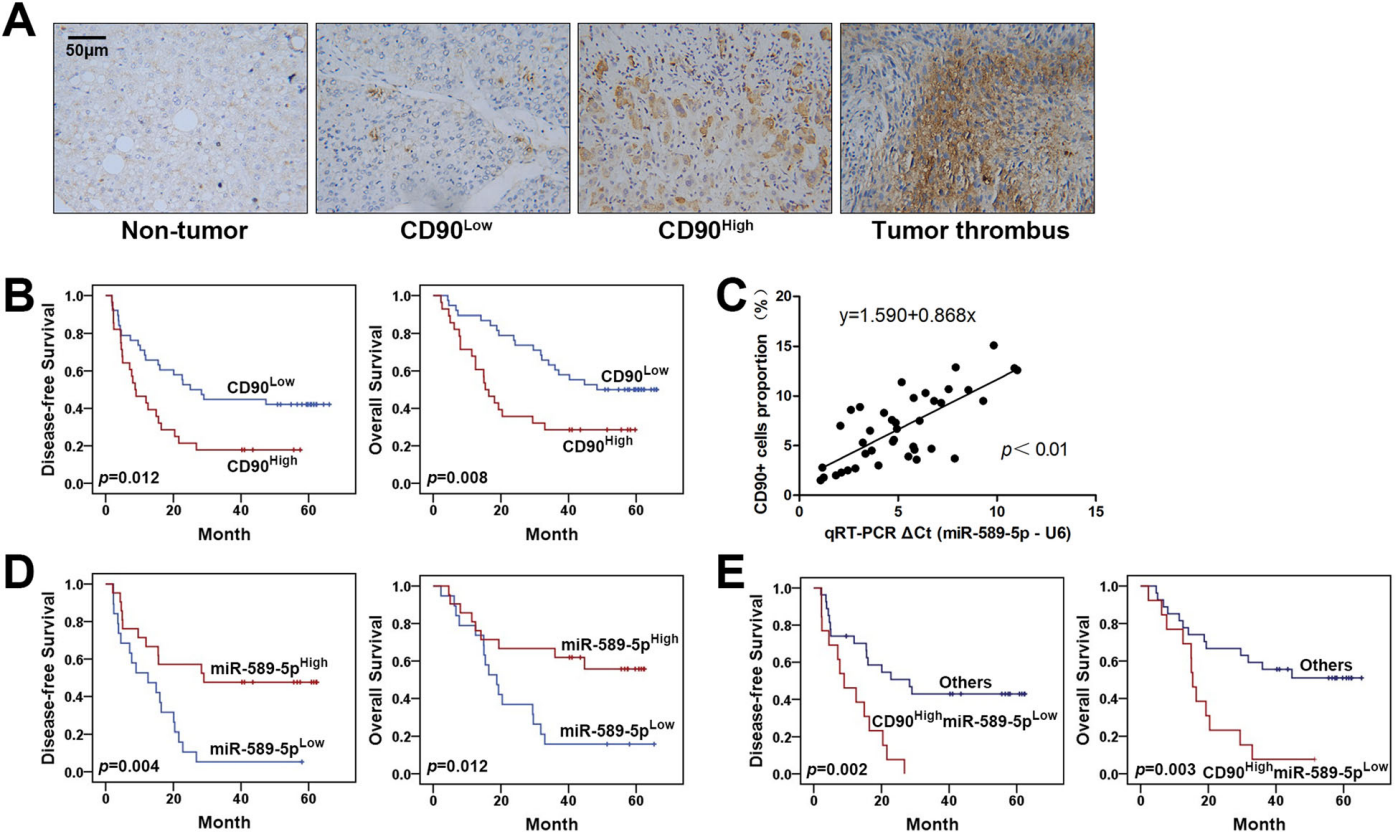
The expression of CD90 and miR-589-5p is associated with a poor clinical prognosis in human HCC. **a** Expression of CD90 in HCC tissues, paired non-tumor tissues and tumor thrombus specimens. **b** Disease-free and overall survival of HCC patients with respect to CD90 expression levels was analyzed by the Kaplan-Meier method. **c** Expression of miR-589-5p and its relationship with CD90 in forty HCC tissue specimens. **d** Disease-free and overall survival of HCC patients with respect to miR-589-5p expression levels was analyzed by the Kaplan-Meier method. **e** Disease-free and overall survival of HCC patients with respect to the combination of CD90 and miR-589-5p expression was analyzed by the Kaplan-Meier method. All assays were performed in triplicate (n = 3)

To evaluate the relationship between CD90 expression and clinical prognosis in human HCC, we detected the expression of CD90 by immunohistochemistry (IHC) in sixty-six tissue samples from HCC patients. As shown in Fig. 5a, CD90 was expressed in all of the non-tumor tissues at a very low basal level. In contrast, only a fraction of the cells in the tumor tissues showed significant positive staining for CD90, ranging from 1.5 to 15.1 %. Samples with over 5 % CD90 staining were considered CD90 high expressers (CD90^High^), and the rest were classified as CD90 low expressers (CD90^Low^). We found that 57.6 % (38/66) of the tumor tissues exhibited low CD90 expression, and 42.4 % (28/66) of the tumor tissues displayed high CD90 expression. Moreover, CD90 expression in the blood vessel thrombi of tumors was higher than in their paired primary tumors. Interestingly, CD90 expression was positively correlated with vascular invasion (*p* < 0.05) and recurrence (*p* < 0.01) (Table 1). Kaplan-Meier survival analysis showed that high expression of CD90 in HCC was associated with significantly decreased disease-free and overall survival (Fig. 5b). Cox regression showed that high expression of CD90 and Edmondson Grade III/IV were independent risk factors of disease-free and overall survival (Table 2). These data suggest that high CD90 expression is associated with a poor prognosis for HCC patients.

**Table 1:** The relationship between CD90 expression and clinical parameters in human HCC (*n* = 66)

Parameters		CD90^Low^	CD90^High^	*p* Value
Male/Female		34/4	26/2	0.969
Age*		52 (32-75)	45.5 (32-60)	0.524
HBsAg				
	Positive	34 (89.5 %)	25 (89.3 %)	1.000
	Negative	4 (10.5 %)	3 (10.7 %)	
AFP				
	Normal	14 (36.8 %)	12 (42.9 %)	0.629
	High	24 (63.2 %)	16 (57.1 %)	
Vascular Invasion				
	Positive	2 (5.3 %)	9 (32.1 %)	0.010
	Negative	36 (94.7 %)	19 (67.9 %)	
Tumer Size(cm)*		4.75 (2-18)	6.5 (3-16)	0.077
Recurrence		21 (55.3 %)	24 (85.7 %)	0.009
Edmondson Grade	I/II	25 (65.8 %)	15 (53.6 %)	0.315
	III/IV	13 (34.2 %)	13 (46.4 %)	

*Value expressed in the midian with the range in parentheses

**Table 2:** Univariate and multivariate analyses of CD90 and other factors associated with survival (*n* = 66)

	DFS		OS	
Variables	HR (95 % CI)	*p* Value	HR (95 % CI)	*p* Value
Univariate analyses				
Gender (male)	0.715 (0.301-1.703)	0.449	0.877 (0.311-2.472)	0.804
Age (≥60y)	0.721 (0.285-1.829)	0.492	0.620 (0.220-1.747)	0.366
HBsAg (positive)	0.619 (0.276-1.389)	0.245	0.728 (0.304-1.740)	0.475
AFP (high)	1.144 (0.629-2.078)	0.659	1.026 (0.542-1.942)	0.937
Vascular Invasion (positive)	2.033 (0.998-4.141)	0.051	1.545 (0.708-3.368)	0.274
Tumer Size(≥5 cm)	2.106 (1.161-3.818)	0.014	2.303 (1.219-4.351)	0.010
Edmondson Grade (III/IV)	6.224 (3.183-12.168)	0.000	3.546 (1.854-6.780)	0.000
CD90^High^	2.680 (1.398-5.135)	0.003	3.072 (1.489-6.337)	0.002
Multivariate analyses				
Tumer Size(≥5 cm)	1.642 (0.887-3.039)	0.114	1.714 (0.898-3.271)	0.102
Edmondson Grade (III/IV)	5.426 (2.746-10.723)	0.000	2.997 (1.555-5.776)	0.001
CD90^High^	1.926 (1.023-3.812)	0.024	2.469 (1.179-5.169)	0.016

Next, we investigated whether there is a relationship between miR-589-5p and CD90 expression in HCC tumor tissues. Of the samples used for IHC staining, forty cases had paired frozen tissues from which RNAs were extracted and assessed for miR-589-5p expression, and we observed that miR-589-5p expression was inversely correlated with CD90 expression (Fig. 5c).

We analyzed the relationship between miR-589-5p expression level and the clinical outcomes of HCC patients and found that those with low miR-589-5p expression had a high risk of vascular invasion (*p* < 0.05) and recurrence (*p* < 0.01) (Table 3). Kaplan-Meier survival analysis showed that low miR-589-5p expression was correlated with low disease-free and overall survival (Fig. 5d). Cox regression showed that low miR-589-5p expression was independent risk factor of disease-free and overall survival (Table 4). These data suggest that miR-589-5p down-regulation in HCC is associated with a poor clinical prognosis.

**Table 3:** The relationship between miR-589-5p expression and clinic parameters in human HCC (*n* = 40)

Parameters		miR-589-5p^Low^	miR-589-5p^High^	*p* Value
Male/Female		15/4	20/1	0.281
Age*		50 (32-65)	48 (32-61)	0.849
HBsAg				
	Positive	15 (78.9 %)	20 (95.2 %)	0.281
	Negative	4 (21.1)	1 (4.8 %)	
AFP				
	Normal	9 (47.4 %)	12 (57.1 %)	0.536
	High	10 (52.6 %)	9 (42.9 %)	
Vascular Invasion				
	Positive	7 (36.8 %)	1 (4.8 %)	0.033
	Negative	12 (63.2 %)	20 (95.2 %)	
Tumer Size(cm)*		7 (4-16)	5.5 (2.5-15)	0.254
Recurrence		18 (94.7 %)	10 (47.6 %)	0.001
Edmondson Grade	I/II	10 (52.6 %)	17 (81.0 %)	0.056
	III/IV	9 (47.4 %)	4 (19.0 %)	

*Value expressed in the midian with the range in parentheses

**Table 4:** Univariate and multivariate analyses of miR-589-5p and other factors associated with survival (*n* = 40)

	DFS		OS	
Variables	HR (95 % CI)	*p* Value	HR (95 % CI)	*p* Value
Univariate analyses				
Gender (male)	0.935 (0.320-2.732)	0.902	0.790 (0.245-2.548)	0.693
Age (≥60y)	0.979 (0.936-1.024)	0.349	1.015 (0.970-1.062)	0.513
HBsAg (positive)	1.041 (0.277-3.916)	0.952	3.165 (0.659-15.196)	0.150
AFP (high)	1.663 (0.718-3.852)	0.235	1.342 (0.530-3.396)	0.535
Vascular Invasion (positive)	1.911 (0.666-5.480)	0.228	1.052 (0.314-3.5181)	0.935
Tumer Size(≥5 cm)	1.157 (1.057-1.266)	0.002	1.190 (1.072-1.321)	0.001
Edmondson Grade (III/IV)	4.350 (1.979-9.561)	0.000	2.975 (1.335-6.631)	0.008
miR-589-5p^Low^	1.269 (1.095-1.470)	0.002	1.226 (1.090-1.469)	0.002
Multivariate analyses				
Tumer Size(≥5 cm)	1.067 (0.949-1.201)	0.279	1.124 (0.984-1.285)	0.085
Edmondson Grade (III/IV)	2.800 (1.140-6.877)	0.025	1.573 (0.648-3.816)	0.317
miR-589-5p^Low^	1.230 (1.028-1.470)	0.023	1.190 (1.002-1.414)	0.048

Because CD90 and miR-589-5p are independent predictors of HCC outcomes, we investigated whether the combination of CD90 and miR-589-5p is a better predictor of an HCC prognosis. We found that HCC patients with CD90^High^miR-589-5p^Low^ expression had a shorter disease-free and overall survival, larger tumor size, and higher risks of vascular invasion and recurrence (Fig. 5e, Additional file 1: Table S4). Therefore, the combination of CD90 and miR-589-5p may be a better predictor of an HCC prognosis.

## Discussion

According to the cancer stem cell theory, CSCs are only a small subset of cells within a tumor, and this population tends to be stable in various environment. An ideal CSC marker should distinguish this subset and be expressed in all primary tumors and cell lines. To date, CD90, CD133 and EpCAM have been used as distinguishing phenotypic markers for enriching HCC CSCs from both primary tumors and cell lines [7–9]. In this study, these potential CSC markers were examined by flow cytometry, and the size of the CD133^+^ and EpCAM^+^ populations varied greatly among the different HCC cell lines. In contrast, CD90 was much more consistently expressed in all of the tested HCC cell lines, ranging from 0.9 % to 3.1 %. In addition, we found that in the cell spheres, the proportion of CD90^+^ cells increased in all cell lines but only up to 11.8 %. Moreover, in every HCC tumor sample examined, only a fraction of the tumor cells showed significant positive staining for CD90, ranging from 1.5-15.1 %. Therefore, CD90 is an ideal CSC marker that is stably expressed in a small cell population.

In HCC, several miRNAs have been shown to regulate CSCs and to play cancer promoting or suppressing roles. It has been reported that exogenous miR-181 increased EpCAM^+^ HCC cell quantity and tumor-initiating ability [19]. In CD133^+^ HCC cells, miR-130b was overexpressed and enhanced chemoresistance, tumorigenicity and self-renewal [18], whereas miR-150 was down-regulated and significantly inhibited tumor sphere formation and cell growth [30]. In this study, we found that miR-589-5p expression was down-regulated in CD90^+^ HCC cells by comparing the miRNA expression profiles of CD90^+^ and CD90^-^ cells, and this result was confirmed by qRT-PCR. Overexpression of miR-589-5p suppressed the CSC characteristics of CD90^+^ HCC cells such as stem cell-associated gene expression (Oct4, Sox2 and Nanog), cell sphere formation, invasiveness and tumorigenicity both *in vitro* and *in vivo*. However, overexpression of miR-589-5p had no impact on the regulation of stemness in CD90^-^ HCC cells, because CD90^-^ HCC cells do not possess CSC characteristics [7]. Moreover, transfection of miR-589-5p antagomir in the whole cell population suppressed the expression of miR-589-5p, but failed to increase CD90^+^ population (Additional file 2: Figure S4). This might due to the low abundance of miR-589-5p in HCC cell lines, antagonizing miR-589-5p did not significantly inhibit miR-589-5p functions. Hence, these data suggest that miR-589-5p is down-regulated in CD90^+^ HCC cells and suppresses stem cell characteristics.

MAP3K8 has been reported to be overexpressed in various human tumors and to promote cell transformation, proliferation, migration, and invasion by activating extracellular signal–regulated kinase (ERK), Rac1, and focal adhesion kinase (FAK) [31, 32]. However, few studies have focused on the role of MAP3K8 in HCC development. One recent study determined that MAP3K8 knockout mice exhibited a significantly lower incidence of liver tumors compared with wild-type mice in diethylnitrosamine-induced tumor formation model [33]. In this study, the *in silico* analysis predicted that MAP3K8 was a potential downstream target of miR-589-5p. Luciferase reporter assays showed that miR-589-5p directly bound to the 3ˈ-UTR of MAP3K8 mRNA, and exogenous miR-589-5p decreased MAP3K8 expression at both the mRNA and protein levels. Moreover, inhibition of MAP3K8 by siRNA significantly reduced the expression of Oct4, Sox2 and Nanog and suppressed self-renewal, migration and invasion. The above findings indicate the importance of MAP3K8 in human HCC tumorigenesis and progression by promoting CD90^+^ CSC stemness characteristics. Overall, miR-589-5p appears to decrease the population of CD90^+^ cells and impair stem cell characteristics partly by silencing MAP3K8.

The status of CSCs might be a key determinant of cancer behavior [34–37]. Our clinical study indicated that the expression levels of CD90 and miR-589-5p were significantly inversely correlated in the HCC clinical specimens, and CD90^+^ HCC samples or samples with decreased miR-589-5p expression showed more vascular invasion and reduced disease-free and overall survival. Moreover, the combination of CD90^High^ and miR-589-5p^Low^ predicted even poorer prognosis. These results might be explained by the high invasive and metastatic capacities of CD90^+^ HCC and the alteration of stemness by miRNAs. Additionally, our *in vivo* study demonstrated that CD90^+^ HCC cells initiate tumor xenografts in immunodeficient mice, whereas CD90^-^ cells and miR-589-5p-transfected CD90^+^ cells do not. One mouse injected with 1 × 10^5^ CD90^-^ cells grew a small tumor by the 11^th^ week, but this tumor xenograft contained CD90^+^ cells (Additional file 2: Figure S5), suggesting that CD90^+^ cells are required to re-establish the cellular hierarchy and to generate tumors in HCC. Thus, CD90 overexpression and miR-589-5p down-regulation indicate more aggressive HCC and poor clinical outcomes.

In summary, the binding of miR-589-5p to the MAP3K8 3ˈ-UTR inhibits MAP3K8 expression and suppresses CD90^+^ CSC characteristics, and the expression status of CD90 and miR-589-5p determines the behavior of HCC. Thus, CD90 and miR-589-5p are useful predictors of HCC progression, and miR-589-5p and MAP3K8 might be novel molecular targets for HCC treatment.

## Conclusions

In HCC, miR-589-5p down-regulates the stemness characteristics of CD90^+^ CSCs in part by silencing MAP3K8. CD90 and miR-589-5p expression predict HCC outcomes and might be novel molecular targets for HCC treatment.

## Additional files

Additional file 1: Table S1. Antibodies used in this study. **Table S2.** The primers have been used for quantitative real-time PCR. **Table S3.** All differentially expressed microRNAs in MHCC97H and MHCC97L CD90^+^ cells. **Table S4.** The relationship of CD90 and miR-589-5p expression to clinical parameters in human HCC (*n*=40). (DOCX 21 kb)
Additional file 2: Figure S1. CD90 is predominantly expressed in a small population in HCC cell lines. **Figure S2.** CD90^+^ HCC cells possess CSC characteristics. **Figure S3.** Overexpression of miR-589-5p has no impact on the regulation of MAP3K8 and stemness in CD90^-^ HCC cells. **Figure S4.** Suppression of miR-589-5p fails to alter the CD90^+^ population in HCC cells. **Figure S5.** CD90^-^ tumor xenograft contains CD90^+^ cells (DOCX 16 kb)

## Abbreviations

bFGF: Basic fibroblast growth factor
COT: Cancer Osaka thyroid
CSCs: Cancer stem cells
DFS: Disease-free survival
EGF: Epidermal growth factor
EpCAM: Epithelial cell adhesion molecule
ERK: Extracellular signal–regulated kinase
FAK: Focal adhesion kinase
HCC: Hepatocellular carcinoma
MAP3K8: Mitogen-activated protein kinase kinase kinase 8
miRNAs: microRNAs
OS: Overall survival
qRT-PCR: quantitative real-time PCR
SEM: Standard error of mean
TICs: Tumor initiating cells
TPL2: Progression locus 2
UTR: Untranslated region
