# Supplementary material for: miR-589-5p inhibits MAP3K8 and suppresses CD90+ cancer stem cells in hepatocellular carcinoma
Source: J Exp Clin Cancer Res. 2016 Nov 11;35:176. doi: 10.1186/s13046-016-0452-6 (PMC5106831; doi:10.1186/s13046-016-0452-6)
Supplement: Additional file 1: Table S1. — Antibodies used in this study. Table S2. The primers have been used for quantitative real-time PCR. Table S3. All differentially expressed microRNAs in MHCC97H and MHCC97L CD90+ cells. Table S4. The relationship of CD90 and miR-589-5p expression to clinical parameters in human HCC (n=40). (DOCX 21 kb) [file 13046_2016_452_MOESM1_ESM.docx]

**Table S1: Antibodies used in this study**

| **Target Protein** | **Vendor** | **Catalog NO.** |
| --- | --- | --- |
| **Antibodies used for FACS and cell sorting** | | |
| CD90 | Biolegend | 328104 |
| EpCAM | Miltenyi Biotec | 130-091-253 |
| CD133 | Miltenyi Biotec | 130-090-854 |
| CD24 | Miltenyi Biotec | 130-095-953 |
| OV-6 | R&D Systems | MAB2020 |
| CD44 | Biolegend | 103033 |
| FITC labeled goat anti-mouse IgG | Zhongshan Glodenbridge | ZF-0312 |
| PE labeled goat anti-rat IgG | Abcam | ab7010 |
| Anti-mouce IgG1 microbeads | Miltenyi Biotec | 130-047-102 |
| **Antibodies used for IHC** | | |
| CD90 | Santa Cruz | sc-9163 |
| **Antibodies used for western blot** | | |
| MAP3K8 | Abcam | ab137589 |
| Oct4 | Abcam | ab19857 |
| Sox2 | Abcam | ab97959 |
| Nanog | Abcam | ab21624 |
| GAPDH | Santa Cruz | sc-25778 |
| HRP labeled goat anti-rabbit IgG | Zhongshan Glodenbridge | ZDR-5306 |

**Table S2: The primers have been used for quantitative real-time PCR**

| **Gene** | **RefSeq accession numbers** | **Primer sequences (5'-3')** |
| --- | --- | --- |
| Nanog | NM_024865.2 | Forward CTCTCCTCTTCCTTCCTCCAT |
|  |  | Reverse TTGCGACACTCTTCTCTGC |
| Oct4 | NM_002701.4 | Forward CCATCTGCCGCTTTGAGG |
|  |  | Reverse ACGAGGGTTTCTGCTTTGC |
| Sox2 | NM_003106.2 | Forward CAGCCCATGCACCGCTACGACG |
|  |  | Reverse CACCGAACCCATGGAGCCAAGAGC |
| MAP3K8 | NM_005204.3 | Forward ATGGAGTACATGAGCACTGGA |
|  |  | Reverse GCTGGCTCTTCACTTGCATAAAG |
| GAPDH | NM_002046.5 | Forward GGAGCGAGATCCCTCCAAAAT |
|  |  | Reverse GGCTGTTGTCATACTTCTCATGG |

**Table S3: All differentially expressed microRNAs in MHCC97H and MHCC97L CD90^+^ cells**

|  | **MHCC97H** | | |  | **MHCC97L** | | |
| --- | --- | --- | --- | --- | --- | --- | --- |
|  | **Name** | **Fold-change** | **miRBase accession numbers** |  | **Name** | **Fold-change** | **miRBase accession numbers** |
| **Up-regulated** | hsa-miR-1246 | 2.605154864 | MIMAT0005898 |  | hsa-miR-208a-3p | 1.500011877 | MIMAT0000241 |
|  | hsa-miR-1290 | 3.821852975 | MIMAT0005880 |  | hsa-miR-1246 | 1.509239736 | MIMAT0005898 |
|  | hsa-miR-149-3p | 1.508759993 | MIMAT0004609 |  | hsa-miR-1290 | 2.017407498 | MIMAT0005880 |
|  | hsa-miR-1827 | 1.535130853 | MIMAT0006767 |  | hsa-miR-183-3p | 1.743972773 | MIMAT0004560 |
|  | hsa-miR-1908-5p | 5.686768282 | MIMAT0007881 |  | hsa-miR-1908-5p | 1.959584237 | MIMAT0007881 |
|  | hsa-miR-21-5p | 1.50128939 | MIMAT0000076 |  | hsa-miR-1915-3p | 1.738359266 | MIMAT0007892 |
|  | hsa-miR-483-3p | 3.144645157 | MIMAT0002173 |  | hsa-miR-1973 | 2.065685771 | MIMAT0009448 |
|  | hsa-miR-494-3p | 1.526976815 | MIMAT0002816 |  | hsa-miR-300 | 1.50045548 | MIMAT0004903 |
|  | hsa-miR-513a-5p | 2.629493781 | MIMAT0002877 |  | hsa-miR-483-3p | 1.633940149 | MIMAT0002173 |
|  | hsa-miR-583 | 2.010995279 | MIMAT0003248 |  | hsa-miR-510-5p | 1.646489569 | MIMAT0002882 |
|  | hsa-miR-765 | 1.932214171 | MIMAT0003945 |  | hsa-miR-583 | 1.97756697 | MIMAT0003248 |
|  |  |  |  |  | hsa-miR-675-5p | 2.211053481 | MIMAT0004284 |
|  |  |  |  |  | hsa-miR-874-3p | 2.292175248 | MIMAT0004911 |
| **Down-regulated** | hsa-miR-141-3p | 0.482836168 | MIMAT0000432 |  | hsa-miR-21-5p | 0.59286745 | MIMAT0000076 |
|  | hsa-miR-1260a | 0.542784652 | MIMAT0005911 |  | hsa-let-7b-5p | 0.449298935 | MIMAT0000063 |
|  | hsa-miR-200c-3p | 0.641339474 | MIMAT0000617 |  | hsa-miR-1260a | 0.194659681 | MIMAT0005911 |
|  | hsa-miR-29a-3p | 0.592854204 | MIMAT0000086 |  | hsa-miR-1264 | 0.277101291 | MIMAT0005791 |
|  | hsa-miR-31-5p | 0.590850576 | MIMAT0000089 |  | hsa-miR-1265 | 0.224445102 | MIMAT0005918 |
|  | hsa-miR-33b-5p | 0.581820193 | MIMAT0003301 |  | hsa-miR-193a-3p | 0.452024745 | MIMAT0000459 |
|  | hsa-miR-487b-3p | 0.516369419 | MIMAT0003180 |  | hsa-miR-215-5p | 0.488413546 | MIMAT0000272 |
|  | hsa-miR-519e-3p | 0.314135766 | MIMAT0002829 |  | hsa-miR-22-3p | 0.356705537 | MIMAT0000077 |
|  | hsa-miR-589-5p | 0.43213539 | MIMAT0004799 |  | hsa-miR-23b-3p | 0.254029295 | MIMAT0000418 |
|  | hsa-miR-891a-5p | 0.610874501 | MIMAT0004902 |  | hsa-miR-33b-5p | 0.213333099 | MIMAT0003301 |
|  |  |  |  |  | hsa-miR-340-5p | 0.52549026 | MIMAT0004692 |
|  |  |  |  |  | hsa-miR-34b-3p | 0.369604064 | MIMAT0004676 |
|  |  |  |  |  | hsa-miR-423-5p | 0.61050992 | MIMAT0004748 |
|  |  |  |  |  | hsa-miR-487b-3p | 0.23160098 | MIMAT0003180 |
|  |  |  |  |  | hsa-miR-519e-3p | 0.104138376 | MIMAT0002829 |
|  |  |  |  |  | hsa-miR-589-5p | 0.216203837 | MIMAT0004799 |
|  |  |  |  |  | hsa-miR-597-5p | 0.358862373 | MIMAT0003265 |
|  |  |  |  |  | hsa-miR-625-3p | 0.640423752 | MIMAT0004808 |
|  |  |  |  |  | hsa-miR-888-3p | 0.338071455 | MIMAT0004917 |
|  |  |  |  |  | hsa-miR-891a-5p | 0.570007242 | MIMAT0004902 |
|  |  |  |  |  | hsa-miR-93-5p | 0.374300239 | MIMAT0000093 |
|  |  |  |  |  | hsa-miR-933 | 0.39009807 | MIMAT0004976 |

**Table S4: The relationship of CD90 and miR-589-5p expression to clinical parameters in human HCC (n=40).**

| **Parameters** | | **CD90^High^ miR-589-5p^Low^** | **Others** | ***p* Value** |
| --- | --- | --- | --- | --- |
| Male/Female |  | 11/2 | 24/3 | 1.000 |
| Age* |  | 50 (32-65) | 48 (32-61) | 0.795 |
| HBsAg |  |  |  |  |
|  | Positive | 10 (76.9%) | 25 (92.6%) | 0.307 |
|  | Negative | 3 (23.1%) | 2 (7.4%) |  |
| AFP |  |  |  |  |
|  | Normal | 6 (46.2%) | 15 (55.6%) | 0.738 |
|  | High | 7 (53.8%) | 12 (44.4%) |  |
| Vascular Invasion |  |  |  |  |
|  | Positive | 6 (46.2%) | 2 (7.4%) | 0.008 |
|  | Negative | 7 (53.8%) | 25 (92.6%) |  |
| Tumer Size(cm)* |  | 9 (4-16) | 5.0 (2.5-15) | 0.034 |
| Recurrence |  | 13 (100.0%) | 15 (55.6%) | 0.004 |
| Edmondson Grade | Ⅰ/Ⅱ | 6 (46.2%) | 21 (77.8%) | 0.045 |
|  | Ⅲ/Ⅳ | 7 (53.8%) | 6 (22.2%) |  |

* Value expressed in midian with range in parentheses
