# Supplementary material for: miR-589-5p inhibits MAP3K8 and suppresses CD90+ cancer stem cells in hepatocellular carcinoma
Source: J Exp Clin Cancer Res. 2016 Nov 11;35:176. doi: 10.1186/s13046-016-0452-6 (PMC5106831; doi:10.1186/s13046-016-0452-6)
Supplement: Additional file 2: Figure S1. — CD90 is predominantly expressed in a small population in HCC cell lines. Figure S2. CD90+ HCC cells possess CSC characteristics. Figure S3. Overexpression of miR-589-5p has no impact on the regulation of MAP3K8 and stemness in CD90- HCC cells. Figure S4. Suppression of miR-589-5p fails to alter the CD90+ population in HCC cells. Figure S5. CD90- tumor xenograft contains CD90+ cells (DOCX 16 kb) [file 13046_2016_452_MOESM2_ESM.docx]

**Supplementary Figure Legends**

**Fig. S1. CD90 is predominantly expressed in a small population in HCC cell lines.**

(A) CD90-, EpCAM-, CD133-, CD24-, OV-6- and CD44-expressing cells were measured in HCC cell lines (MHCC97H, MHCC97L, HepG2 and SMMC-7721) using flow cytometry. Compared to other CSC markers, CD90 is expressed in a small population in all of the HCC cell lines.

(B) The HCC cell lines were cultured for sphere formation, and the formed spheres were dissociated into single cells to assess the CD90-expressing cells using flow cytometry. The proportion of CD90^+^ cells remained low in all of the cell lines.

All assays were performed in triplicate (n=3).

**Fig. S2. CD90^+^ HCC cells possess CSC characteristics.**

(A) Expression of the pluripotency-associated genes Oct4, Sox2 and Nanog in the sorted CD90^+^ and CD90^-^ MHCC97H cells was measured by qRT-PCR (left and middle panels) and Western blot (right panel).

(B) Sphere formation and clone formation by CD90^+^ and CD90^-^ HCC cells.

(C) Migration and invasion of CD90^+^ and CD90^-^ HCC cells.

(D) Tumor formation by CD90^+^ and CD90^-^ HCC cells in nude mice.

All data are representative of three independent experiments and are shown as mean ± SEM (n=3).

**Fig. S3. Overexpression of miR-589-5p has no impact on the regulation of MAP3K8 and stemness in CD90^-^ HCC cells.**

(A) Expression of miR-589-5p increased in CD90^-^ MHCC97H and MHCC97L cells after transfection with miR-control or miR-589-5p mimics.

(B) Expression of the pluripotency-associated genes Oct4, Sox2 and Nanog in CD90^-^ MHCC97H and MHCC97L cells was measured by qRT-PCR (left and middle panels) and Western blot (right panel) after transfection of miR-control or miR-589-5p mimics.

(C) Sphere formation and clone formation by CD90^-^ MHCC97H and MHCC97L cells transfected with miR-control or miR-589-5p mimics.

(D) Migration and invasion of CD90^-^ MHCC97H and MHCC97L cells transfected with miR-control or miR-589-5p mimics.

(E) CD90^-^ MHCC97H and MHCC97L cells were transfected with miR-589-5p mimics for 24 hours, and the level of MAP3K8 expression was measured by qRT-PCR and Western blot.

All data are representative of three independent experiments and are shown as mean ± SEM (n=3).

**Fig. S4. Suppression of miR-589-5p fails to alter the CD90^+^ population in HCC cells**

(A) Transfection of miR-589-5p antagomir inhibits miR-589-5p expression in MHCC97H and MHCC97L cell lines.

(B) No significant change of CD90^+^ population in MHCC97H and MHCC97L cell lines measured by flow cytometry after transfection of miR-589-5p antagomir.

All assays were performed in triplicate (n=3).

**Fig. S5. CD90^-^ tumor xenograft contains CD90^+^ cells**

(A) Expression of CD90 in CD90^-^ tumor xenograft as determined by IHC.

(B) CD90-expressing cells in CD90^-^ tumor xenograft measured using flow cytometry.

All assays were performed in triplicate (n=3).
